# AdcBC-dependent zinc uptake influences physiological responses in *Streptococcus mutans*

**DOI:** 10.1080/20002297.2026.2691469

**Published:** 2026-06-25

**Authors:** Marlus da Silva Pedrosa, Charles Stephen Schuls, Leonardo Luca Iannaccone, Apoena Aguiar Ribeiro

**Affiliations:** a Department of Diagnostic Sciences (Cariology and Microbiology), Adams School of Dentistry, University of North Carolina at Chapel Hill, Chapel Hill, NC, USA; b Department of Orofacial Sciences, School of Dentistry, University of California, San Francisco, CA, USA; c College of Arts and Sciences, University of North Carolina at Chapel Hill, Chapel Hill, NC, USA

**Keywords:** *Streptococcus mutans*, zinc, AdcBC, biofilm, EPS, acidogenicity, dental caries, metal homeostasis

## Abstract

**Background:**

Zinc is widely used in oral care products due to its antimicrobial and anti-biofilm properties; however, the molecular mechanisms by which zinc influences *Streptococcus mutans* (*S. mutans*) physiology remain incompletely understood. The AdcABC system is the primary high-affinity zinc transporter in *S. mutans*. We investigated whether loss of *adcBC* is associated with altered physiological responses in *S. mutans*.

**Material and methods:**

Wild-type (WT), Δ*adcBC* mutant, and complemented *S. mutans* UA159 strains were evaluated under zinc-replete and zinc-limited conditions using zinc sulphate (ZnSO_4_) at defined concentrations. Growth, carbohydrate utilization, acidogenicity, acid tolerance, aggregation, biofilm formation, and expression of stress response and regulatory genes were assessed.

**Results:**

Loss of *adcBC* impaired growth and reduced utilization of multiple carbohydrates, including key glycolytic substrates, indicating altered metabolism. Zinc supplementation restored growth but did not consistently recover redox-based metabolic activity. Expression of *pflA*, associated with fermentative metabolism, was reduced in the mutant. Acid tolerance was unaffected by AdcBC. Zinc enhanced aggregation and surface attachment independently of AdcBC, whereas biofilm biomass showed partial dependence on AdcBC-mediated zinc uptake. The Δ*adcBC* mutant also exhibited altered expression of genes involved in oxidative stress, metal homeostasis, and regulatory pathways.

**Conclusion:**

Loss of *adcBC* is associated with altered physiological responses in *S. mutans*. Zinc influences bacterial physiology through both uptake-dependent and uptake-independent mechanisms, highlighting its context-dependent role in oral environments.

## Introduction

Dental caries remains one of the most widespread chronic non-communicable conditions globally, affecting over 2 billion individuals and ranking as the most prevalent health issue [1]. It is a highly prevalent biofilm-mediated disease characterised by the demineralisation of tooth enamel and dentin due to acid production by bacterial communities [2]. It arises from the dysbiosis of the oral microbiota, where frequent sugar intake and prolonged low pH selectively enrich acidogenic and aciduric species [2–4]. Among these, *Streptococcus mutans* (*S. mutans*) is a key contributor due to its remarkable ability to ferment dietary carbohydrates into organic acids (acidogenicity), tolerate low pH (aciduricity), synthesise extracellular polysaccharides (EPS) that enhance biofilm integrity, and engage in genetic competence and stress adaptation [2,5–8]. These traits enable *S. mutans* to thrive in the acidic microenvironment of carious lesions and make it a key target for preventive strategies [2,9].

**Figure 1.**
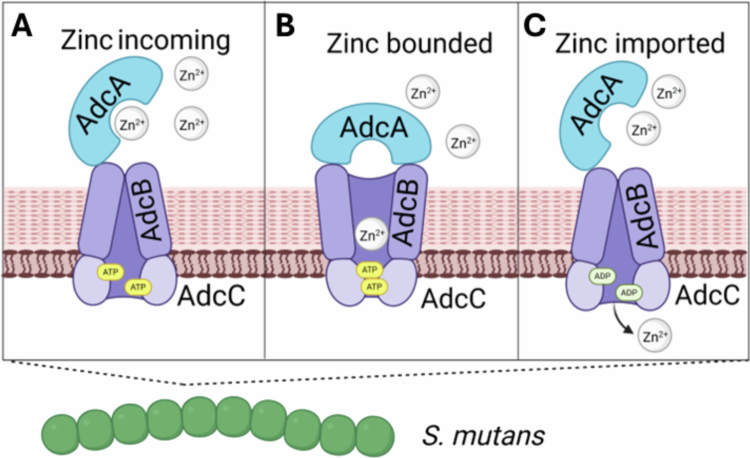
Schematic representation of the AdcABC zinc uptake system in *S. mutans*. The AdcABC system is an ABC-type high-affinity zinc uptake transporter, in which AdcA functions as the zinc-binding lipoprotein, AdcB as the transmembrane permease, and AdcC as the cytoplasmic ATPase. This schematic is based on previously described mechanisms of AdcABC-mediated zinc transport in streptococci [11,14,15,47,48]. Created in BioRender. PEDROSA, M. (2026) https://BioRender.com/5h371ug. (A) AdcA, located on the bacterial surface, captures extracellular zinc and delivers it to the transporter. (B) AdcB forms the transport channel, facilitating zinc translocation across the membrane. (C) AdcC provides energy for zinc transport through ATP hydrolysis.

Zinc is an essential micronutrient for both microbial and host physiology [10]. Bacteria have evolved sophisticated homoeostatic systems to maintain intracellular zinc balance, involving both uptake under zinc limitation and efflux-mediated detoxification under zinc-replete conditions [11]. In Gram-positive bacteria, zinc detoxification is mediated by efflux systems that export excess intracellular zinc; In *S. mutans*, excess intracellular zinc is exported by the *P*-type ATPase ZccE, which prevents zinc toxicity and contributes to metal homoeostasis [12]. In parallel, bacteria rely on uptake systems to acquire zinc when availability is limited. In Gram-positive bacteria, including streptococci such as *S. mutans*, the AdcABC ATP-binding cassette (ABC) transporter represents the primary high-affinity zinc uptake system (Figure 1) [13–16]. Although AdcABC is the primary high-affinity zinc uptake system, additional low-affinity or non-specific pathways may contribute to zinc acquisition under zinc-replete conditions.

Zinc (Zn^2+^) is a naturally present trace metal in the oral cavity, where it plays dual roles as an essential micronutrient and a potential antimicrobial agent. Physiological Zn^2+^ concentrations in saliva typically range from 0.0002 to 0.005 mM, while dental biofilms may retain higher levels, between ~0.098 mM and 0.612 mM [17]. These concentrations are generally sufficient to support bacterial survival, metabolic activity, and stress resistance in *S. mutans*. However, oral care products, including toothpaste and mouth rinses, can contain up to 1% w/w zinc, often in the form of zinc sulphate or zinc citrate, corresponding to approximately 35 mM Zn^2+^ [18]. Upon brushing with a standard amount of toothpaste (~0.25 g), an estimated 0.57 mg of zinc may be introduced into the oral cavity, of which 15–40% may be transiently retained on oral surfaces [17]. This retention may result in local zinc concentrations of approximately 0.08–0.22 mM, overlapping with the upper range of physiological levels found in dental biofilms.

Despite its importance, relatively few studies have examined how zinc uptake systems in *S. mutans* influence virulence-associated traits [14]. This question is particularly relevant because zinc is both a naturally occurring trace element in the oral cavity and a widely used additive in oral health care products (e.g. toothpastes and mouthwashes) for its antimicrobial and anti-biofilm properties [17,19–26].

In these applications, zinc is commonly delivered in the form of soluble salts such as zinc sulphate (ZnSO_4_), zinc chloride (ZnCl_2_), or zinc citrate [17,19–26]. Previous studies have shown that zinc can inhibit the growth of *S. mutans*, reduce biofilm formation, and interfere with enzymatic processes involved in carbohydrate metabolism and acid production, although these effects are concentration-dependent [17,19–26]. The biological response of *S. mutans* to zinc has been extensively characterised, particularly regarding its effects on growth, metabolic activity, and biofilm-associated behaviours [17,19–31]. In contrast, the mechanistic basis of these effects remains incompletely understood, particularly with respect to the relative contributions of AdcBC-mediated zinc uptake [14,15,32].

This study investigated the role of the AdcBC high-affinity zinc uptake system in regulating metabolic and virulence-associated traits in *S. mutans*. Using the UA159 wild-type (WT) strain and an isogenic Δ*adcBC* mutant, we evaluated the impact of zinc uptake on growth, carbohydrate utilisation, acidogenicity, acid tolerance, aggregation, and biofilm formation on hydroxyapatite (HA) surfaces. In addition, we assessed the expression of genes involved in oxidative stress response (*sod*, *dpr*), metal homoeostasis (*sloR*), and regulatory pathways associated with competence and biofilm development (*comX*, *vicR*, *dexA*) to define how intracellular zinc uptake influences transcriptional networks. While previous studies have primarily focused on the role of zinc transport in growth and metal homoeostasis [14,15,32], this study further examines AdcBC-dependent zinc uptake in *S. mutans* by integrating metabolic profiling, phenotypic characterisation, and gene expression analysis to assess how zinc availability influences bacterial physiology under different conditions.

## Material and methods

### Bacterial strains and overnight culture

*S. mutans* UA159 (serotype c), a proven virulent cariogenic dental pathogen, was used in all experiments. An *S. mutans* UA159 mutant strain that lacks the zinc import system AdcC ATPase and AdcB permease (Δ*adcBC*), and a complement strain rescuing the zinc import system (Δ*adcBC*_*compl*_), were generated as previously reported [14]. Bacteria were routinely cultured on BBL™ Trypticase™ Soy Agar with 5% Sheep Blood (TSA II, BD, Cat.# 221261). For liquid culture, a single colony was inoculated into Brain Heart Infusion (BHI) broth (Thermo Scientific™ Oxoid™, Cat.# CM1135B) and incubated at 37 °C in 5% CO_2_. To support growth of the Δ*adcBC* mutant under zinc-limited conditions, BHI was supplemented with 10 µM ZnSO_4_, representing the minimal concentration required to enable reliable growth and obtain sufficient biomass for downstream assays [14].

### Growth assays

Growth assays were performed as described elsewhere [33]. Briefly, overnight cultures were centrifuged and washed three times with 1 × phosphate-buffered saline (PBS) to remove any trace compounds in the culture media. Cells were resuspended and normalised by optical density at 600 nm (OD_600_) in test media (OD_600_ = 0.1). Culture were grown at 37 °C and 5% CO_2_ without shaking. The OD_600_ was recorded every 1 h using a microplate reader (SpectraMax M2e). Specific growth conditions included varying concentrations of zinc or other chemical treatments, as indicated in the corresponding figure legends.

### Zinc conditions

Zinc-limited conditions were generated using the zinc-specific chelator N, N, N′, N′-tetrakis(2-pyridylmethyl)ethylenediamine (TPEN; Sigma-Aldrich, Cat.# P4413), which was used at a final concentration of 10 µM to reduce bioavailable zinc in the growth media. Untreated conditions correspond to standard BHI or TSB without additional supplementation. BHI and TSB are complex media that contain trace amounts of zinc and were therefore considered zinc-replete conditions relative to TPEN-treated (zinc-limited) conditions [12,34].

Where indicated, zinc supplementation was performed using ZnSO_4_. For growth curve analyses, a range of 0.01, 0.1, 1, and 10 mM ZnSO_4_ was used to assess dose-dependent effects. For all other experiments, including carbohydrate utilisation, acid production, acid tolerance, aggregation, biofilm formation, and gene expression analyses (RT-qPCR), ZnSO_4_ was used at a final concentration of 0.1 mM, unless otherwise specified. This concentration was selected as it falls within physiologically relevant ranges in oral cavity (~0.08–0.22 mM) [17,19–23] and represents the highest concentration tested that did not inhibit bacterial growth. In addition, alternative zinc salts, including zinc chloride (ZnCl_2_; Sigma-Aldrich, Cat.# 208086) and zinc acetate (Zn(CH_3_COO)_2_; Sigma-Aldrich, Cat.# 383058), were used in selected experiments (Figure S1) to confirm that observed growth trends were not specific to a single zinc formulation.

### Carbohydrate utilisation assay using PrebioM™ plates

To assess the impact of zinc on carbohydrate metabolism in *S. mutans*, we used the PreBioM™ Mono/Disaccharide Prebiotic Substrate Utilisation Assay (BIOLOG Inc., Cat.# F-0001) [33,35,36], which contains 95 individual carbon sources, including sugars, sugar alcohols, and glycosides. Both wild-type (WT) and Δ*adcBC* mutant strains were tested.

A single colony of each strain was inoculated into 5 mL of BHI and grown overnight at 37 °C under microaerophilic conditions (5% CO_2_). As detailed in the Bacterial Strains and Culture section, overnight cultures were supplemented with 10 µM ZnSO₄ to support growth of the Δ*adcBC* mutant strain. Cultures were then diluted 1:20 in fresh BHI and grown to mid-log phase, followed by centrifugation at 4000 × g for 5 minutes. Cell pellets were washed twice with Dulbecco’s Phosphate-Buffered Saline (DPBS) without calcium and magnesium (Thermo Fisher Scientific™, Cat.# 14190144) and resuspended in carbon-free inoculating fluid (IF-0a GN/GP, BIOLOG Inc., Cat.# 72268). The inoculating fluid was supplemented with Redox Dye Mix A (BIOLOG Inc., Cat.# 74221) at a final concentration of 1× (from a 100× stock). Bacterial suspensions were either left untreated or supplemented with ZnSO_4_ at a final concentration of 0.1 mM, representing zinc-replete conditions. Untreated conditions did not include additional zinc supplementation; however, all cultures had prior exposure to 10 µM ZnSO_4_ during overnight growth, which was consistent across conditions.

A total of 100 µL of the suspension was added to each well of the PreBioM™ plate. Plates were incubated at 37 °C under ambient air. Metabolic activity was monitored as an 8 h time-course with absorbance readings collected at regular intervals, and the data presented correspond to the 8 h endpoint. The assay is based on substrate utilisation in microplates preloaded with defined carbon and energy sources; when bacteria metabolise a given substrate, cellular redox reactions lead to the reduction of Redox Dye Mix A, resulting in colour development. This signal reflects cumulative metabolic (redox) activity rather than turbidity-based growth, enabling detection of substrate-specific metabolic utilisation independent of biomass accumulation [35,36].

Following incubation, metabolic activity was quantified by measuring dye reduction at an optical density (OD) of 590 nm using a microplate reader. Absorbance values were normalised to the average of negative control wells (no carbon source). Each condition was performed in biological triplicate, with each biological replicate measured in technical duplicate, and representative results from three independent experiments are shown.

### Assessment of acid production and acid tolerance

*S. mutans* strains were inoculated at an initial OD_600_ of 0.1 in TSB, and acid production was monitored by measuring the pH of the culture supernatant over time using a benchtop pH metre (FiveEasy F20, Mettler Toledo), following established protocols [37]. A decline in pH was used as an indicator of acid production and fermentation activity. To assess acid tolerance [33,37], BHI was adjusted to pH 5.0, 6.0, or 7.0 by dropwise addition of L-lactic acid (Sigma-Aldrich, Cat.# 252476) under continuous monitoring with a calibrated pH metre, rather than by targeting a predefined molar concentration. While mineral acids are commonly used for pH adjustment, L-lactic acid was selected to better reflect physiologically relevant conditions. Cultures were normalised to OD_600_ = 0.1 and inoculated into the pH-adjusted media. For each condition, 200 μL of culture was transferred to a 96-well plate and growth was monitored by OD_600_ readings taken every hour over time using a microplate reader (SpectraMax M2e). Growth differences across pH conditions were used to evaluate the acid resistance of each strain. Each condition was tested in three independent biological experiments, each performed in triplicate with technical duplicates.

### Bacterial aggregation assay

Bacterial aggregation was assessed using an OD_600_-based protocol previously described [33,38]. Briefly, overnight cultures of *S. mutans* WT and Δ*adcBC* were diluted to an OD_600_ of 0.1 in BHI broth supplemented with or without ZnSO₄ (0.1 mM final concentration). Cultures (5 mL) were incubated statically in sterile borosilicate Ki-max test tubes at 37 °C with 5% CO_2_ for 24 h. Following incubation, tubes were gently inverted to disrupt loose cell attachment and vortexed briefly (10 seconds) to resuspend aggregated cells. This approach is consistent with previously described OD-based method [33,38] used to monitor aggregation behaviour in liquid cultures; however, in the absence of direct visualisation or sedimentation measurements, OD_600_ values primarily reflect total biomass following resuspension and do not provide a direct quantitative measure of aggregation. Accordingly, results are interpreted as reflecting aggregation-associated or surface-associated behaviour rather than aggregation alone. All conditions were tested in biological quintuplicate (*n* = 5), and OD values were normalised to the untreated WT control.

### Biofilm formation on hydroxyapatite (HA) discs

Monospecies biofilms of *S. mutans* were established using BHI broth supplemented with 0.2% sucrose (BHI-sucrose) to promote biofilm development as previously described [33]. HA discs (7 mm × 1.8 mm; HiMed Inc.), used as a tooth enamel analogue, were UV-sterilised before use. Overnight cultures of *S. mutans* grew in BHI at 37 °C in 5% CO_2_. Cultures were diluted in fresh BHI-sucrose to an OD_600_ of 0.1, and 1 mL of inoculum was added to each well of a sterile 48-well flat-bottom polystyrene plate, with one saliva-coated HA disc mounted in a vertical position per well. Plates were incubated at 37 °C in 5% CO_2_. At 24 h, HA discs were gently rinsed with PBS to remove non-adherent cells and used for downstream analyses. Each condition was tested in three independent biological experiments, each performed in triplicate with technical duplicates.

### Biofilm biomass quantification by crystal violet

To quantify total biofilm biomass, discs were transferred to new 48-well plates and stained with 0.1% crystal violet solution for 15 minutes at RT. Excess stains were removed by washing the wells or discs 2 × with sterile water to remove planktonic and loosely bound bacteria, and adherent (biofilm) cells stained with 0.1% crystal violet for 15 min. The bound dye was eluted in a 33% acetic acid solution, and the total biofilm was estimated by measuring the absorbance of the dissolved dye at 575 nm [14,33].

### Colony forming unit (CFU) enumeration

Following completion of experimental treatments, cultures were serially diluted in sterile phosphate-buffered saline (PBS) and plated on BHI agar. For the Δ*adcBC* mutant, BHI agar plates were supplemented with 10 µM ZnSO_4_ to support growth. Plates were incubated at 37 °C in 5% CO_2_ for 48 h, and colony-forming units (CFUs) were counted to determine CFU/mL. For samples grown on HA discs, each disc was placed in a sterile tube containing 1 mL PBS and vortexed for 30 seconds to dislodge surface-associated bacteria. The resulting suspension was serially diluted, and 7 µL aliquots of each dilution were plated onto BHI agar. To calculate CFU per unit area (CFU/cm^2^), colony counts were multiplied by the dilution factor and divided by the plated volume (in mL) and the total surface area of the HA disc (1.1654 cm^2^), accounting for all exposed surfaces.

### Total exopolysaccharide (EPS) quantification

Total extracellular polysaccharides (EPS) produced by *S. mutans* biofilms were quantified using the phenol–sulphuric acid method, which measures carbohydrate content via furfural derivative formation and chromogenic reaction with phenol [33,39,40]. Biofilms were cultivated on HA discs as described above. After 24 h of incubation, each disc was transferred to a sterile 1.5 mL microcentrifuge tube containing 350 μL of 1 M NaOH, then vortexed vigorously for 30–60 seconds to dislodge the biofilm. Samples were incubated at 37 °C for 3 h and centrifuged at 10,000 × g for 10 minutes to separate solubilized EPS. Later, 50 μL of the resulting supernatant or glucose standard (ranging from 12.5 to 800 μg/mL) was transferred to a 96-well plate. Reactions were initiated by adding 150 μL of concentrated sulphuric acid (95–98%; Sigma-Aldrich, # 258105) followed by 30 μL of 5% phenol solution (w/v; Sigma-Aldrich, Cat# P1037). After incubation at RT for 10 minutes, absorbance was measured at 490 nm using a microplate reader (SpectraMax M2e). A glucose standard curve (Sigma, Cat# G8270) was used to calculate EPS concentration, expressed as µg glucose equivalents per sample. Each condition was tested in three independent biological experiments, each performed in triplicate with technical duplicates.

### Bacterial RNA extraction

*S. mutans* strains were cultured as described above. RNA extraction and downstream analyses, including cDNA synthesis and RT-qPCR, were performed following a previously described protocol [33]. Overnight cultures were centrifuged, and the bacterial pellets were resuspended in fresh BHI. Optical density was normalised to OD_600_ = 0.1, and cultures were either left untreated or treated with zinc (0.1 mM) for 8 h. Briefly, bacterial pellets were harvested by centrifugation and resuspended in freshly prepared lysozyme solution (10 mg/mL; 100 µL per sample), followed by the addition of 1 µL of 10% SDS. Samples were then processed using a homogeniser equipped with single-use cartridges (Invitrogen, Cat. #12183026) to ensure thorough mechanical disruption. RNA was subsequently purified using the PureLink™ RNA Mini Kit (Invitrogen, Cat.# 12183025), including on-column DNase I treatment (PureLink DNase Set, Invitrogen, Cat.# 12185010) to remove genomic DNA contamination, following the manufacturer’s protocol. RNA was eluted in RNase-free water and quantified using a NanoDrop™ spectrophotometer to assess concentration and integrity.

### cDNA synthesis

First-strand cDNA synthesis was performed using the High-Capacity cDNA Reverse Transcription Kit with RNase Inhibitor (Applied Biosystems, Cat# 43-688-14). For each reaction, 0.5 µg of total RNA was used in a 20 µL reaction volume, following the manufacturer’s protocol. Synthesised cDNA was diluted 10-fold (20 µL cDNA + 180 µL water) before RT-qPCR.

### Reverse Transcription Quantitative PCR (RT-qPCR)

Reverse Transcription Quantitative PCR was performed using SYBR™ Green PCR Master Mix (Applied Biosystems, Cat.# 4309155) in 384-well optical plates (Applied Biosystems, Cat# AB1384). Each 10 µL reaction contained 5 µL SYBR Green Master Mix, 0.5 µL each of forward and reverse primers, 2 µL nuclease-free water, and 2 µL of diluted cDNA template. Full primer sequences and the corresponding source references [41–46] are provided in Table 1. No-template controls (NTCs) were included for every primer pair. Thermocycling conditions consisted of an initial denaturation at 95 °C for 10 minutes, followed by 40 cycles of 95 °C for 15 seconds and 60 °C for 60 seconds. Ct values were obtained using default threshold settings. Relative gene expression was calculated using the ∆∆Ct method with 16S rRNA as the internal reference gene. Expression levels were normalised to the untreated wild-type control group. RT-qPCR analyses were performed on three independent biological replicates, each measured in technical duplicate.

**Table 1.** Primer sequences and functional roles of target genes used in RT-qPCR analysis.

Target gene	Function	F/R	Sequence 5’-3’	Sequence reference
*16S rRNA*	Housekeeping gene (reference)	F	CACACCGCCCGTCACACC	[41]
		R	CAGCCGCACCTTCCGATACG	
*comX*	Alternative sigma factor regulating late competence genes; key regulator in quorum sensing and genetic transformation	F	CGTCAGCAAGAAAGTCAGAAAC	[41]
		R	ATACCGCCACTTGACAAACAG	
*vicR*	Response regulator of the VicRK two-component system; regulates genes related to cell wall metabolism, stress response, and biofilm development	F	CGCAGTGGCTGAGGAAAATG	[43]
		R	ACCTGTGTGTGTCGCTAAGTGATG	
*dexA*	Encodes a debranching glucan hydrolase; breaks down branched glucans, contributing to biofilm remodelling and dispersal	F	AGGGCTGACTGCTTCTGGAGT	[43]
		R	AGTGCCAAGACTGACGCTTTG	
*sloR*	A key metalloregulator that coordinates responses to manganese, iron, and zinc. It regulates multiple virulence genes. sloR is highly responsive to changes in metal availability.	F	CGTCATCTCTTTATCGCAAGCATC	[44]
		R	CAGTATGTTCCAGCACTTCAGCC	
*dpr*	Protects DNA from oxidative damage and is regulated by metal stress	F	TGGTTCAGGCTTCCTTTATCTGC	[44]
		R	CTTCCTCATCTGTCACATCAAGACC	
*pflA*	Encodes pyruvate formate-lyase-activating enzyme; involved in anaerobic energy metabolism and fermentation.	F	CCTGGTCTGACTGATCGGGATG	[45]
		R	TGTTGGCGGTTTAACACCTTCC	
*sod*	Superoxide dismutase; detoxifies superoxide radicals into hydrogen peroxide, critical for oxidative stress tolerance	F	GTTTTGGCTCAGGTTGGGCT	[46]
		R	ATAGTTTGGACGAACATTAC	

F, forward sequence; R, reverse sequence.

### Statistical analysis

Normal data distribution was verified using the Shapiro–Wilk test. Statistical analyses were conducted in GraphPad Prism version 11 (GraphPad Software, CA, USA), with a significance level set at 5% (*α* = 0.05). The specific statistical tests applied are detailed in the corresponding figure legends. Significance is indicated as follows: *P* < 0.05 (*)*, P < 0.01* (**)*, P < 0.001* (***), and *P* < 0.0001 (****).

## Results

### AdcBC contributes to growth under zinc-limited conditions and modulates the response to zinc availability in S. mutans

**Figure 2.**
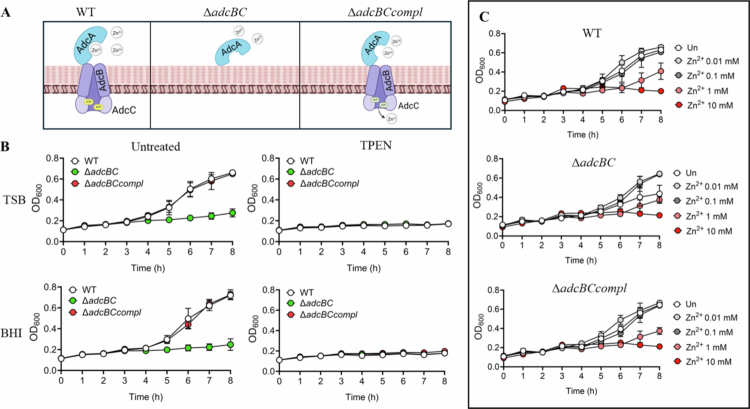
AdcBC contributes to growth under zinc-limited conditions and modulates the response to zinc availability in *S. mutans*. Growth of *S. mutans* UA159 wild-type (WT), Δ*adcBC* mutant, and complemented strain (Δ*adcBC*_*compl*_) under conditions of varying zinc availability. (**A**) Schematic representation of the AdcABC zinc uptake system, including the solute-binding protein AdcA, membrane permease AdcB, and ATPase AdcC. Created in BioRender. PEDROSA, M. (2026) https://BioRender.com/016nrcw. (**B**) Growth curves in TSB (top panels) and BHI (bottom panels) under untreated conditions (left panels; no additional zinc supplementation) and TPEN-treated conditions (right panels; 10 µM TPEN). Untreated conditions correspond to standard media containing baseline zinc levels present in complex media, whereas TPEN treatment reduces bioavailable zinc through chelation. Under TPEN-treated conditions, growth was markedly reduced in all strains relative to untreated conditions. (**C**) Growth curves in BHI supplemented with increasing concentrations of ZnSO₄ (0.01, 0.1, 1, and 10 mM). Un, untreated (baseline zinc conditions without additional supplementation). Data represent mean ± SD from three independent biological replicates, each performed in technical triplicate.

To determine the contribution of the AdcBC zinc uptake system to *S. mutans* physiology, we compared the growth kinetics of UA159 wild-type (WT), the Δ*adcBC* mutant, and the complemented strain (Δ*adcBC*_compl_) (Figure 2A) under conditions of varying zinc availability.

Under untreated conditions (no additional zinc supplementation), the Δ*adcBC* mutant exhibited reduced growth compared to the WT and complemented strains in both TSB and BHI, as reflected by lower biomass accumulation and delayed entry into exponential phase (Figure 2B, left panels). Complementation restored growth to levels comparable to WT, confirming that this phenotype is attributable to loss of *adcB* and *adcC* [14]. Under TPEN-treated conditions (10 µM), which reduce zinc availability [14], growth was markedly reduced in all strains relative to untreated conditions (Figure 2B, right panels), indicating that zinc limitation broadly impaired bacterial growth under these conditions.

We next assessed how increasing concentrations of exogenous zinc affect bacterial growth (Figure 2C). At lower concentrations (0.01–0.1 mM), zinc supported growth across all strains. However, higher concentrations (1–10 mM) resulted in growth inhibition in all strains, consistent with zinc toxicity at elevated levels. No consistent differences in zinc sensitivity were observed between strains at higher concentrations. Consistent growth trends were observed when alternative zinc salts were tested (Figure S1), indicating that these effects are not specific to ZnSO_4_.

Together, these findings indicate that AdcBC contributes to optimal growth under zinc-limited conditions, while growth can be maintained in the presence of baseline or supplemented zinc, and that zinc availability influences bacterial growth in a dose-dependent manner.

### Loss of adcBC is associated with altered carbohydrate utilisation and metabolic gene expression in S. mutans

**Figure 3.**
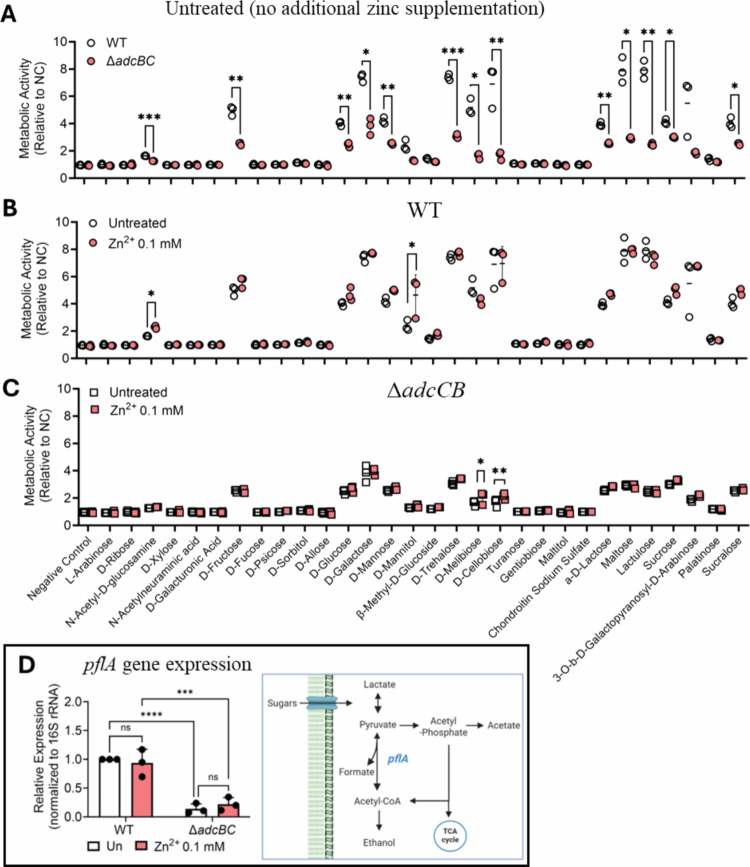
Loss of *adcBC* is associated with altered carbohydrate utilisation and gene expression in *S. mutans*. Metabolic activity and gene expression of *S. mutans* UA159 wild-type (WT) and Δ*adcBC* mutant strains under untreated and zinc-supplemented conditions. Metabolic activity was assessed using a redox-based assay, which does not necessarily correlate with biomass accumulation or fermentation output. Untreated WT and Δ*adcBC* values shown in panel A are reused in panels B and C for direct comparison and represent the same biological replicates. Untreated conditions correspond to baseline zinc present in complex media (no additional zinc supplementation). (**A**) Metabolic activity of WT and Δ*adcBC* strains across a panel of carbon sources under untreated conditions, measured using the PreBioM™ substrate utilisation assay. Data are expressed as relative metabolic activity normalised to negative control wells (NC). The Δ*adcBC* mutant exhibited reduced utilisation of multiple substrates compared to WT. (**B**) Metabolic activity of WT in the presence or absence of Zn²⁺ (0.1 mM). Zinc supplementation produced minimal changes across most substrates. (**C**) Metabolic activity of the Δ*adcBC* mutant in the presence or absence of Zn^2+^ (0.1 mM). Zinc supplementation did not restore metabolic activity across the substrates tested. (**D**) Relative expression of *pflA* measured by RT-qPCR and normalised to 16S rRNA. Expression levels are shown relative to untreated WT. The Δ*adcBC* mutant exhibited significantly reduced *pflA* expression compared to WT, and zinc supplementation did not restore expression. A schematic of fermentative pathways highlighting the role of *pflA* is shown below. Created in BioRender. PEDROSA, M. (2026) https://BioRender.com/cvnpl1c. Data represent mean ± SD from three independent biological replicates, with technical duplicates averaged prior to analysis. Statistical analysis for panels A–C was performed using multiple t-tests with Holm–Šídák correction for multiple comparisons, while panel D was analysed using two-way ANOVA with appropriate post-hoc comparisons. Significance is indicated as *P < 0.05, **P < 0.01, and ***P < 0.001.

Given that carbohydrate utilisation is central to *S. mutans* virulence, driving acid production and biofilm development, and that zinc serves as a cofactor for numerous metabolic enzymes, we next assessed whether loss of *adcBC* affects the ability of *S. mutans* to utilise diverse carbon sources. Metabolic activity of WT and Δ*adcBC* strains was evaluated using the PreBioM™ substrate utilisation platform, which includes a panel of mono- and disaccharides relevant to the oral environment (Figure 3A–C). The PreBioM™ assay provides an indirect measure of redox-based metabolic activity, which does not necessarily correlate with biomass accumulation or fermentation output. Based on the dose–response growth curves (Figure 2C), 0.1 mM Zn^2+^ was used for subsequent experiments, as it represents the highest concentration that supports growth without inhibitory effects.

Under untreated conditions, the Δ*adcBC* mutant exhibited markedly reduced metabolic activity across multiple substrates compared to WT, including glycolytic sugars, sugar alcohols, and disaccharides (Figure 3A). To determine whether extracellular zinc could compensate for this defect, we evaluated substrate utilisation in the presence of Zn^2+^ (0.1 mM). In WT, zinc supplementation produced minimal changes in metabolic activity across most substrates (Figure 3B). Similarly, zinc supplementation did not substantially alter metabolic activity across most substrates in the Δ*adcBC* mutant (Figure 3C).

To further assess whether the observed defects in carbohydrate utilisation extend to central fermentative pathways, we examined the expression of *pflA*, which encodes pyruvate formate-lyase, a key enzyme in anaerobic metabolism that converts pyruvate into formate and acetyl-CoA during mixed-acid fermentation [49]. This pathway lies directly downstream of glycolytic carbohydrate processing and is essential for energy production under conditions relevant to oral biofilms (Figure 3D, bottom panel). Consistent with the impaired utilisation of multiple carbon sources observed in the Δ*adcBC* mutant (Figure 3A–C), *pflA* expression was significantly reduced compared to WT (*p* < 0.0001), consistent with altered expression of a gene associated with fermentative metabolism (Figure 3D, top panel). Notably, zinc supplementation did not restore *pflA* expression, mirroring the lack of rescue observed in the substrate utilisation assays.

Together, these findings indicate that the Δ*adcBC* mutant exhibits reduced carbohydrate utilisation across multiple substrates and decreased expression of *pflA*, a gene associated with fermentative metabolism. The inability of extracellular zinc supplementation to restore *pflA* expression under the conditions tested suggests that this phenotype is not readily rescued by zinc supplementation alone. However, because the complemented strain was not evaluated in this assay and intracellular zinc levels were not directly measured, these findings should be interpreted as phenotypes associated with loss of *adcBC* rather than definitive evidence of impaired zinc-dependent metabolic flux.

### Acidogenic potential in S. mutans is associated with zinc-supported growth

**Figure 4.**
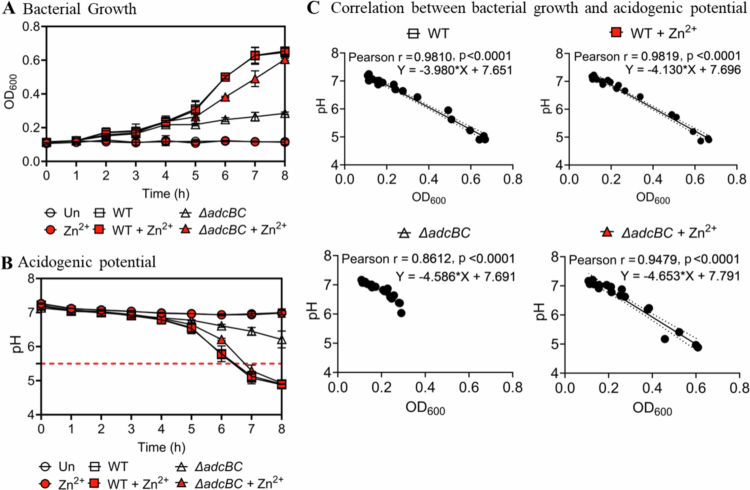
Zinc-supported growth is associated with acidogenic potential in *S. mutans*. (**A**). Growth curves of *S. mutans* UA159 wild-type (WT), Δ*adcBC*, and complemented strain (Δ*adcBC*_compl_) in BHI with and without Zn^2+^ (0.1 mM ZnSO_4_). (**B**). Acidogenic potential of *S. mutans* UA159 WT, Δ*adcBC*, and Δ*adcBCcompl* in BHI supplemented Zn^2+^ (0.1 mM ZnSO_4_), measured as pH over time. (**C**). Pearson correlation analysis between bacterial growth (OD_600_; panel A) and acidogenic potential (pH; panel B). All experiments were performed independently three times, each in duplicate. Data represent mean ± SD from three independent biological experiments, with technical duplicates averaged prior to analysis. Statistical significance for correlation analysis was determined using Pearson correlation.

Given that carbohydrate metabolism underlies acid production and cariogenicity in *S. mutans*, and that zinc supplementation failed to restore carbohydrate utilisation in the Δ*adcBC* mutant under growth-independent conditions, we next examined whether these defects impact acidogenic potential. Bacterial growth (OD_600_) and medium acidification (pH) were monitored over time in WT, Δ*adcBC*, and complemented strains, with or without ZnSO_4_ (0.1 mM) supplementation (Figure 4A-B).

As expected, the Δ*adcBC* mutant exhibited reduced growth and maintained a near-neutral pH, indicating minimal acid production. In contrast, WT and the complemented strain showed robust growth accompanied by progressive acidification to approximately pH 5. Zinc supplementation restored both growth and acidification in the Δ*adcBC* mutant to levels comparable to WT. Across all strains and conditions, bacterial growth and medium acidification were strongly correlated (Pearson correlation, *p* < 0.0001; Figure 4C), indicating that acid production is closely linked to bacterial growth under the conditions tested. Notably, this contrasts with the carbohydrate utilisation assays, in which bacterial growth was not permitted and zinc supplementation failed to rescue metabolic activity in the Δ*adcBC* mutant.

Together, these findings indicate that acidogenic potential in *S. mutans* is not solely dependent on AdcBC-mediated zinc uptake, but instead reflects growth-dependent effects supported by zinc availability. The inability of zinc to restore carbohydrate utilisation under growth-independent conditions suggests that intracellular zinc uptake is required for metabolic flexibility, whereas acid production can be recovered indirectly through restoration of growth.

### Acid tolerance in S. mutans is independent of AdcBC-mediated zinc uptake

**Figure 5.**
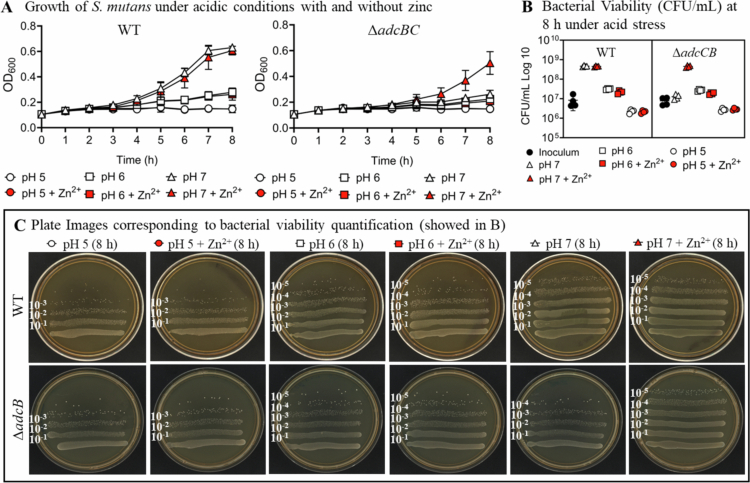
Acid tolerance in *S. mutans*is largely independent of AdcBC-mediated zinc uptake. Effect of pH on growth and viability of *S. mutans* UA159 wild-type (WT) and Δ*adcBC* mutant strains in the presence or absence of Zn^2+^ (0.1 mM ZnSO_4_). pH adjustments were performed using lactic acid. (**A**) Growth of WT and Δ*adcBC* strains across pH 7, 6, and 5, with and without zinc supplementation. (**B**) Viability measured as colony-forming units (CFU/mL) under the same conditions. (**C**) Representative images of culture plates used for CFU enumeration. Data in (**A-B**) represent mean ± SD from three independent biological experiments, with technical duplicates averaged prior to analysis.

S. mutans thrives in acidic environments and contributes to caries pathogenesis by outcompeting less acid-tolerant commensals. Given that zinc supplementation restored growth and acid production in the Δ*adcBC* mutant (Figure 4), despite failing to rescue carbohydrate utilisation under growth-independent conditions (Figure 3), we next examined whether AdcBC-mediated zinc uptake contributes to acid tolerance. Growth of WT and Δ*adcBC* strains was assessed in BHI adjusted to pH 7, 6, or 5, with or without 0.1 mM Zn^2+^ supplementation (Figure 5).

As expected, acidic pH impaired growth in both strains, with OD_600_ values decreasing progressively from pH 7 to pH 5. While no statistically significant differences were observed between WT and Δ*adcBC* under acidic conditions (pH 6 and 5), a modest reduction in CFU was observed in the Δ*adcBC* mutant at pH 7 under untreated conditions, consistent with its reduced growth in the absence of sufficient zinc availability. Zinc supplementation did not improve growth at low pH in either strain. Consistent with these findings, bacterial viability (CFU/mL) decreased at lower pH in both strains, with no consistent genotype-dependent differences observed under acidic conditions (Figure 5B-C). To assess whether lactate itself influenced growth independently of pH, we evaluated the effect of L-lactate supplementation (10 and 25 mM) and observed no significant differences in growth (Figure S2), indicating that the observed effects are more consistent with pH-dependent conditions rather than lactate-specific effects.

These results indicate that acid tolerance in *S. mutans* is independent of AdcBC-mediated zinc uptake and is not enhanced by extracellular zinc. In contrast to acid production, which reflects zinc-supported growth, survival under acidic stress appears to be governed by mechanisms that are less dependent on intracellular zinc acquisition under the conditions tested.

### Zinc enhances aggregation and surface attachment while partially supporting biofilm biomass in S. mutans

**Figure 6.**
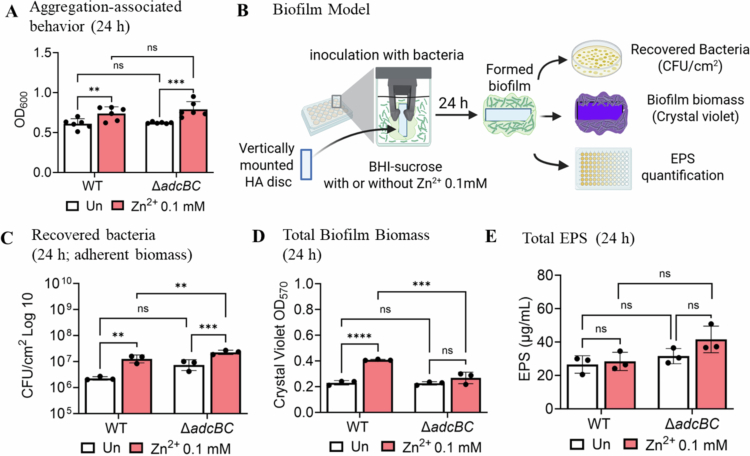
Zinc influences aggregation-associated and surface-associated phenotypes while partially supporting biofilm biomass in *S. mutans*. Aggregation and biofilm formation of *S. mutans* UA159 wild-type (WT) and Δ*adcBC* mutant strains in the presence or absence of Zn^2+^ (0.1 mM ZnSO_4_). Aggregation and biofilm formation were assessed following 24 h incubation under the indicated conditions. For aggregation-associated behaviour, cultures were incubated statically and OD_600_ was measured after resuspension. For biofilm assays, bacteria were grown on hydroxyapatite (HA) discs in BHI supplemented with sucrose, followed by quantification of viable adherent bacteria, total biofilm biomass, and extracellular polysaccharides. (**A**) Aggregation-associated behaviour after 24 h, measured as OD_600_ following resuspension. OD_600_ values reflect culture density and cell–cell interactions rather than direct quantification of aggregation. (**B**) Schematic representation of the hydroxyapatite (HA) disc biofilm model. Bacteria were grown in BHI supplemented with sucrose for 24 h with or without Zn^2+^ (0.1 mM), followed by quantification of adherent bacteria, total biofilm biomass, and extracellular polysaccharides. Created in BioRender. PEDROSA, M. (2026) https://BioRender.com/gg4tsdf. (**C**) Viable bacteria recovered from HA discs after 24 h, expressed as CFU/cm^2^ (log10), representing adherent biomass following incubation rather than direct measurement of initial attachment. (**D**) Total biofilm biomass on HA discs after 24 h, quantified by crystal violet staining (OD_570_). (**E**) Total extracellular polysaccharide (EPS) production associated with HA biofilms after 24 h, expressed as μg/mL. Data represent mean ± SD from three independent biological experiments, with technical duplicates averaged prior to analysis. Statistical analysis was performed using two-way ANOVA with Tukey’s multiple comparisons test. Significance is indicated as ns (not significant), **P < 0.01, ***P < 0.001, and ****P < 0.0001.

We next examined whether zinc influences aggregation-associated and surface-associated phenotypes in *S. mutans*, and whether these effects depend on AdcBC-mediated zinc uptake. After 24 h, supplementation with 0.1 mM Zn^2+^ significantly increased OD_600_ values following resuspension in both WT and Δ*adcBC* strains (WT, *p* < 0.01; Δ*adcBC*, *p* < 0.001), with comparable baseline values observed between strains under untreated conditions (Figure 6A). Given the assay conditions, these measurements reflect changes in culture density and aggregation-associated behaviour rather than direct quantification of aggregation.

To evaluate these effects under more physiologically relevant conditions, biofilm formation was assessed using a hydroxyapatite (HA) disc model (Figure 6B). Initial inoculum levels were normalised between strains prior to incubation (Figure 6C). After 24 h, zinc supplementation significantly increased the number of viable adherent bacteria recovered from HA discs in both WT and Δ*adcBC* strains (WT, *p* < 0.01; Δ*adcBC*, *p* < 0.001), reflecting viable adherent biomass following incubation rather than direct measurement of initial attachment (Figure 6C).

Despite similar increases in attachment, zinc differentially affected biofilm biomass. In WT, zinc supplementation significantly increased total biofilm biomass (*p* < 0.0001), whereas no significant change was observed in the Δ*adcBC* mutant (Figure 6D). In contrast, total EPS production remained unchanged across all conditions (Figure 6E), indicating that zinc-dependent effects on biofilm biomass are not driven by changes in overall polysaccharide production.

Together, these findings indicate that zinc promotes modest, context-dependent changes in aggregation-associated and surface-associated phenotypes, while biofilm biomass formation is partially dependent on intracellular zinc acquisition.

### AdcBC-mediated zinc uptake influences stress response and regulatory gene expression in S. mutans

To determine how zinc availability and AdcBC-mediated uptake influence transcriptional regulation in *S. mutans*, we quantified the expression of genes involved in oxidative stress response, metal homoeostasis, and regulatory pathways associated with competence and biofilm development. WT and Δ*adcBC* strains were grown in BHI with or without 0.1 mM Zn^2+^ for 8 h, and gene expression was measured by RT-qPCR and normalised to 16S rRNA.

**Figure 7.**
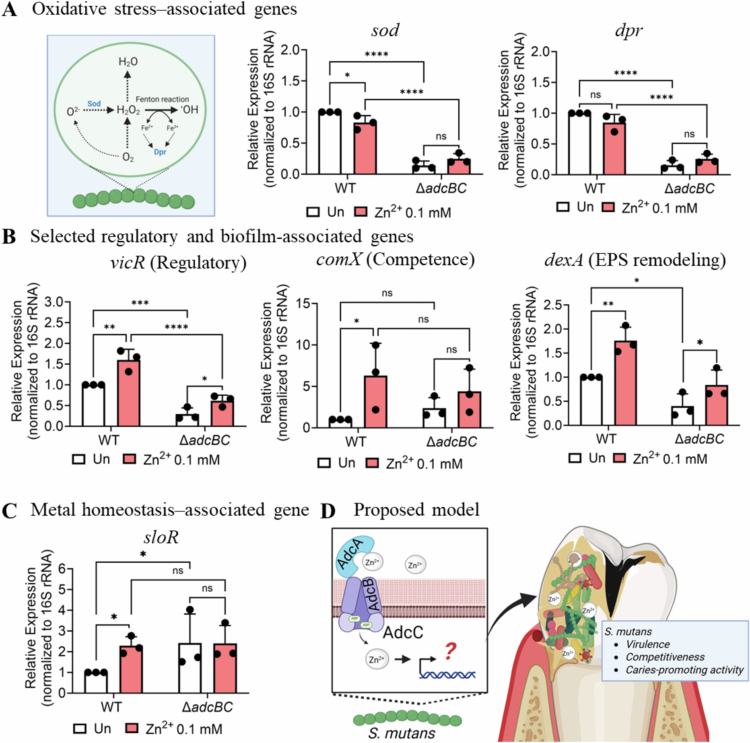
Loss of *adcBC* is associated with altered stress response and regulatory gene expression in *S. mutans*. Gene expression was analysed by RT-qPCR in S. mutans UA159 wild-type (WT) and Δ*adcBC* mutant strains grown in BHI for 8 h in the presence or absence of Zn²⁺ (0.1 mM). Expression levels were normalised to 16S rRNA and are presented relative to untreated WT. (**A**) Oxidative stress response. A schematic (left) illustrates pathways involving Sod and Dpr. Created in BioRender. PEDROSA, M. (2026) https://BioRender.com/l4jmpgh. Relative expression of *sod* (middle) and *dpr* (right) is shown for WT and Δ*adcBC* strains. (**B**) Regulatory and biofilm-associated genes. Relative expression of *vicR* (left), *comX* (middle), and *dexA* (right) is shown under untreated and zinc-supplemented conditions. (**C**) Metal homoeostasis. Relative expression of *sloR* is shown for WT and Δ*adcBC* strains with and without zinc supplementation. (**D)** Schematic illustrating a proposed model for how AdcBC-mediated zinc uptake may influence intracellular regulatory pathways associated with stress response and biofilm-related functions in *S. mutans*. Created in BioRender. PEDROSA, M. (2026) https://BioRender.com/kunro8r. Data represent mean ± SD from three independent biological replicates, with technical duplicates averaged prior to analysis. Statistical analysis was performed using two-way ANOVA with Tukey’s multiple comparisons test. Significance is indicated as ns (not significant), *P < 0.05, **P < 0.01, ***P < 0.001, and ****P < 0.0001.

Genes associated with oxidative stress defence were strongly affected by loss of AdcBC. Expression of *sod*, encoding superoxide dismutase [50,51], and *dpr*, involved in peroxide stress resistance [46], was significantly higher in WT compared to the Δ*adcBC* mutant (*p* < 0.0001 for both), with minimal response to zinc supplementation in either strain (Figure 7A). These findings are consistent with a reduced capacity for oxidative stress response in the absence of efficient intracellular zinc uptake.

Regulatory pathways associated with biofilm development and adaptation were also altered (Figure 7B). Expression of *vicR*, a response regulator involved in cell wall homoeostasis and biofilm formation [43,52,53], was reduced in the Δ*adcBC* mutant compared to WT (*p* < 0.001) and increased in response to zinc in both strains. Expression of *comX*, a sigma factor controlling the competence regulon [6,7] was comparable between strains under untreated conditions but was significantly induced by zinc in WT (*p* < 0.05) and not in the Δ*adcBC* mutant (Figure 7B). Similarly, *dexA*, encoding a dextranase involved in EPS remodelling [34], was expressed at higher levels in WT than in the mutant and was further induced by zinc in both strains (WT *p* < 0.01; Δ*adcBC* *p* < 0.05), although expression remained lower in the mutant.

Consistent with disruption of metal homoeostasis, expression of *sloR*, a key metalloregulator controlling metal ion homoeostasis [54], differed between strains (Figure 7C). Under untreated conditions, *sloR* expression was elevated in the Δ*adcBC* mutant relative to WT (*p* < 0.05). Zinc supplementation induced *sloR* expression in WT (*p* < 0.05) but had no significant effect in the mutant, indicating disrupted metal-responsive regulation when intracellular zinc uptake is impaired.

Together, these findings are consistent with a role for AdcBC in intracellular zinc homoeostasis and associated regulatory responses (Figure 7D). This model links the observed transcriptional changes with the phenotypic effects described above, although direct measurement of intracellular zinc levels would be required to confirm this relationship.

## Discussion

Zinc homoeostasis is critical for maintaining bacterial viability and virulence, influencing processes well beyond simple metal acquisition, including metabolic activity, stress adaptation, and gene regulation [10,11,14,15]. In this study, we investigated the role of the AdcBC high-affinity zinc transporter in *S. mutans*, demonstrating its contribution to growth under zinc-limited conditions, carbohydrate metabolism, and transcriptional regulation. Loss of *adcBC* impaired the utilisation of key dietary sugars (Figure 3A–C) and altered the expression of stress-related and regulatory genes (Figure 7), supporting a role for intracellular zinc in coordinating metabolic and regulatory networks required for survival in the oral cavity. In contrast, zinc-dependent increases in aggregation and surface attachment occurred independently of AdcBC-mediated uptake (Figure 6), indicating that zinc modulates *S. mutans* physiology through both intracellular and extracellular mechanisms. Consistent with prior work demonstrating the role of AdcBC in growth and zinc homoeostasis [14,15,32], our findings extend this framework by integrating metabolic profiling, transcriptional analyses, and phenotypic characterisation to assess how zinc availability influences multiple aspects of *S. mutans* physiology. Together, these findings underscore the context-dependent effects of zinc on oral pathogens and raise important considerations for the formulation of oral care products, where zinc exposure may differentially influence bacterial phenotypes.

Our results demonstrate that AdcBC contributes to growth under zinc-limited conditions rather than being strictly required under all conditions. Under TPEN-treated conditions, which reduce zinc availability, growth of all strains was diminished relative to untreated conditions, indicating that zinc availability broadly limits growth under these conditions. Given that TPEN may exert off-target effects at higher concentrations, these findings should be interpreted as reflecting reduced zinc availability rather than complete zinc deprivation. Accordingly, these results are best explained by limitation of bioavailable zinc rather than a signalling role of extracellular zinc. Although rescue experiments combining TPEN with exogenous zinc were not performed in this study, previous work has demonstrated that zinc supplementation can restore growth under TPEN-induced zinc limitation, supporting the interpretation that TPEN primarily acts by reducing bioavailable zinc [55].

Importantly, under untreated conditions, the Δ*adcBC* mutant retained the ability to grow, indicating that baseline zinc levels present in complex media such as BHI and TSB are sufficient to support growth in the absence of AdcBC-mediated uptake. This observation is consistent with previous findings in *S. mutans*, where Adc-deficient strains maintain growth in the presence of micromolar zinc concentrations [14,32]. Zinc supplementation further supported growth across strains, reinforcing the role of zinc availability as a key determinant of bacterial proliferation. The ability of zinc supplementation to restore growth in the Δ*adcBC* mutant further indicates that zinc can enter the cell via AdcBC-independent mechanisms when extracellular concentrations are sufficient.

Together, these findings indicate that AdcBC functions as the primary high-affinity zinc uptake system required under zinc-limited conditions, while growth can be sustained under zinc-replete conditions, likely through alternative low-affinity or non-specific uptake pathways. Although such pathways have not been fully characterised in *S. mutans*, similar redundancy in metal acquisition systems has been described in other bacteria.

At higher concentrations, zinc inhibited growth in all strains, consistent with its toxic effects at elevated levels [10,11,47,56]. Excess zinc can disrupt metal homoeostasis, interfere with metalloprotein function, and promote oxidative stress [10,11,47]. In *S. mutans*, resistance to zinc toxicity is mediated in part by the ZccE efflux system, which facilitates zinc detoxification and protects against intracellular metal overload [12]. This system likely acts in concert with AdcBC-mediated uptake to maintain intracellular zinc within a narrow physiological range, balancing acquisition and detoxification. These findings align with previous reports in streptococci demonstrating the importance of AdcBC-mediated zinc acquisition for growth under zinc-restricted conditions [14,15,32,57], while also highlighting the need for coordinated uptake and efflux mechanisms to sustain zinc homoeostasis under fluctuating environmental conditions.

While some studies have demonstrated beneficial effects of zinc on cariogenic bacteria and biofilms *in vitro* and *in vivo* [14,58], its impact on *S. mutans* growth remains context-dependent and influenced by concentration, formulation, and bioavailability [26,31,58,59]. Our findings support this view, showing that zinc promotes growth at physiologically relevant concentrations but becomes inhibitory at higher levels (Figure 2). Importantly, although extracellular zinc supported growth under certain conditions, it did not fully restore metabolic activity in the Δ*adcBC* mutant (Figure 3), indicating that intracellular uptake contributes to metabolic function. In contrast, zinc-dependent effects on acid production and biofilm-associated phenotypes were largely linked to growth and surface interactions rather than direct metabolic rescue (Figures 4 and 6). These observations highlight the importance of carefully balancing zinc levels in oral health formulations.

Consistent with the growth-dependent effects of zinc described above, the Δ*adcBC* mutant exhibited broad defects in the utilisation of key carbohydrates, including D-glucose, D-fructose, D-galactose, and D-mannose (Figure 3A–C), indicating that intracellular zinc contributes to sustaining central metabolic pathways. These sugars feed directly into glycolysis and downstream fermentation, and their impaired utilisation suggests disruption of core metabolic flux through central carbon metabolism [60]. The PreBioM™ assay used in this study reflects redox-based metabolic activity and may not directly correspond to growth or acid production, which can proceed through alternative metabolic pathways. The observed defects in glycolytic substrates are consistent with altered metabolic activity and may reflect changes in metabolic processes associated with zinc availability. Supporting this interpretation, expression of *pflA* (Figure 3D), which encodes pyruvate formate-lyase involved in anaerobic fermentative metabolism [49] was significantly reduced in the Δ*adcBC* mutant and was not fully restored by zinc supplementation. The incomplete restoration of metabolic and transcriptional phenotypes despite growth recovery suggests that intracellular zinc-dependent processes may not be fully re-established under these conditions. Given that carbon flux in *S. mutans* can proceed through alternative pathways such as lactate dehydrogenase, reduced *pflA* expression does not necessarily indicate complete impairment of metabolism. Together, these results suggest that extracellular zinc alone may be insufficient to fully restore all metabolic phenotypes evaluated in this study.

These metabolic defects were further reflected in functional phenotypes. Acid production, a key cariogenic trait [8,61], was reduced in the Δ*adcBC* mutant but restored by zinc supplementation (Figure 4), consistent with recovery of growth rather than direct restoration of metabolic capacity. In contrast, acid tolerance [62] remained unaffected by loss of AdcBC (Figure 5), indicating that survival under acidic conditions is independent of zinc uptake. Together, these findings demonstrate that AdcBC-mediated zinc uptake contributes to metabolic flexibility and indirectly supports acidogenic potential through its role in sustaining growth, while being dispensable for basal resistance to acid stress.

Despite these metabolic impairments, zinc significantly enhanced aggregation-associated and surface-associated phenotypes in both WT and Δ*adcBC* strains (Figure 6), indicating that these effects are largely independent of AdcBC-mediated uptake. Given the assay conditions, these measurements reflect changes in culture density and cell–cell interactions rather than direct quantification of aggregation. In the hydroxyapatite (HA) model, zinc supplementation increased the number of viable bacteria recovered after 24 h, reflecting adherent biomass following incubation rather than direct measurement of initial attachment, as growth occurs during this period. In contrast, biofilm biomass increased in WT but not in the Δ*adcBC* mutant, suggesting that biofilm maturation may be partially dependent on intracellular zinc acquisition. Notably, total EPS production remained unchanged across conditions, indicating that zinc-dependent effects on biofilm formation are not driven by bulk polysaccharide synthesis but may instead reflect changes in cell–surface interactions or matrix organisation. Importantly, zinc-dependent effects on aggregation-associated behaviour were most apparent at elevated zinc concentrations, and no strong defect was observed in the Δ*adcBC* mutant under baseline conditions, suggesting that these effects are modest and context-dependent.

Transcriptional profiling further supports this distinction between intracellular and extracellular zinc effects. Genes involved in oxidative stress defence, including *sod* and *dpr* [46,50,51], were significantly downregulated in the Δ*adcBC* mutant (Figure 7A), indicating impaired capacity to detoxify reactive oxygen species. Expression of *sloR*, a key metalloregulator controlling metal homoeostasis [54], was dysregulated in the mutant and showed limited responsiveness to zinc, consistent with disrupted intracellular metal sensing (Figure 7C). The elevated baseline expression of *sloR* observed in the Δ*adcBC* mutant may represent a compensatory response to altered intracellular metal homoeostasis, reflecting disruption of zinc-dependent regulatory networks. In addition, regulatory genes associated with competence and biofilm adaptation, including *comX* [7,8,63], *vicR* [53,64], and *dexA* [8,65], exhibited altered expression patterns in the absence of AdcBC (Figure 7B), indicating that intracellular zinc uptake is required for coordinated activation of these pathways. However, this analysis is based on a targeted set of genes and does not represent a comprehensive transcriptomic assessment of zinc-dependent regulation. Accordingly, these findings should be interpreted as indicative of altered regulatory responses rather than definitive evidence of pathway-wide changes. Together, these data are consistent with a role for AdcBC in intracellular zinc homoeostasis and associated regulatory response (Figure 7D), which may influence phenotypes associated with competitiveness, virulence, and cariogenic potential in *S. mutans* [14]. This model reflects observed phenotypic and transcriptional trends but requires further validation using broader transcriptomic approaches, direct measurements of intracellular zinc, and inclusion of complemented strains in future studies. Because the complemented strain was not included in all assays, these findings should be interpreted as associations observed in the Δ*adcBC* mutant rather than definitive evidence of direct AdcBC-dependent regulation.

This study presents some limitations. The experiments were performed using complex growth media (BHI and TSB), which are not chemically defined and may contain variable baseline levels of zinc and other metals [14]. Zinc concentrations in these media were not directly measured in this study, and therefore absolute zinc availability may vary across conditions. Although this could influence baseline zinc exposure, similar phenotypic trends were consistently observed, supporting the robustness of the findings. The use of these media provides a physiologically relevant context for modeling bacterial behaviour and is appropriate for informing future studies incorporating zinc supplementation in polymicrobial and *in vivo* systems. Moreover, while our data identify clear associations between intracellular zinc uptake and transcriptional regulation, the specific molecular mechanisms by which zinc influences these regulatory networks remain to be defined.

In addition, intracellular zinc levels were not directly measured in this study, and thus the relationship between zinc availability and gene expression is inferred from phenotypic and transcriptional responses. Furthermore, the complemented strain was not included in all experimental assays, which limits confirmation of phenotype specificity across all conditions. Although key phenotypes were validated in growth assays, future studies should include the complemented strain in metabolic, biofilm, and gene expression analyses. It is also important to note that zinc was evaluated as an isolated factor, whereas in oral care formulations, it is typically combined with other bioactive compounds [66] and in alternative forms such as zinc chloride or zinc acetate, which may differ in solubility and bioavailability [18,19,26]. We observed similar growth curve patterns across these zinc salts (Figure S1), suggesting that the effects on *S. mutans* growth are not specific to a single formulation. However, potential differences in biofilm formation and gene regulation between zinc species, as well as interactions with other formulation components, were not systematically assessed and warrant further investigation.

Additionally, the use of TPEN to induce zinc-limited conditions may introduce off-target effects, and therefore observed phenotypes should be interpreted as reflecting reduced zinc availability rather than complete zinc deprivation, as previously discussed in studies using TPEN to model zinc restriction [55]. Rescue experiments combining TPEN with exogenous zinc supplementation were not performed, and thus the reversibility of TPEN-mediated effects was not directly assessed. Furthermore, pH adjustments were performed using lactic acid, which may introduce physiological effects beyond acidification, particularly given the role of organic acids in *S. mutans* metabolism and acid adaptation [67]. Alternative acids (e.g. mineral acids) were not evaluated and may yield different responses.

Finally, while this study focuses on the role of AdcBC-mediated zinc uptake, other components of zinc homoeostasis were not directly evaluated. In particular, the ZccE efflux system, which contributes to zinc detoxification in *S. mutans* [12], was not assessed in this study. Given that zinc homoeostasis depends on the coordinated balance between uptake and efflux, the absence of ZccE functional analyses limits our ability to fully define how intracellular zinc levels are regulated under conditions of zinc excess. Studies examining the interplay between AdcBC-mediated uptake and ZccE-mediated efflux will be important to better understand how *S. mutans* maintains zinc homoeostasis and adapts to fluctuating metal availability.

Future studies incorporating multispecies biofilm models, host–microbe interactions, and *in vivo* systems will be essential to determine how zinc uptake systems influence microbial competition, biofilm architecture, and disease progression under physiologically relevant conditions. Such approaches will also help clarify how zinc-containing oral care products can be optimised to balance antimicrobial efficacy with preservation of microbial homoeostasis.

## Conclusion

This study demonstrates that loss of *adcBC* is associated with altered metabolic activity and gene expression patterns in *S. mutans*. While extracellular zinc supported aggregation-associated and surface-associated phenotypes, it did not consistently restore carbohydrate utilisation, suggesting that AdcBC may contribute to optimal metabolic function. Biofilm biomass was also altered in association with loss of *adcBC*, although the underlying mechanisms require further investigation.

Together, these findings establish zinc as both a nutrient and an environmental factor influencing *S. mutans* physiology in a context-dependent manner. These results have implications for zinc-containing oral care products and suggest that modulating zinc availability or uptake pathways may represent a potential strategy for influencing cariogenic traits.

## Supplementary Material

Supplementary Material Supplementary File.docx
