# Supplementary material for: AdcBC-dependent zinc uptake influences physiological responses in Streptococcus mutans
Source: J Oral Microbiol. 2026 Jun 25;18(1):2691469. doi: 10.1080/20002297.2026.2691469 (PMC13307382; doi:10.1080/20002297.2026.2691469)

**Supplementary File**

Fig. S1

**Growth of *S. mutans* in the presence of alternative zinc salts.**
Growth curves of *S. mutans* UA159 wild-type (WT), Δ*adcBC* mutant, and complemented strain (Δ*adcBC*_compl_) in TSB (top panels) and BHI (bottom panels) supplemented with zinc chloride (left panels) or zinc acetate (right panels) (0.01 mM). Bacterial growth was monitored over time by measuring optical density at 600 nm (OD_600_). Similar growth trends were observed across zinc formulations, indicating that the effects on *S. mutans* growth are not specific to ZnSO_4_ as shown in Fig. 2C). Data represent mean ± SD from three independent biological experiments, with technical duplicates averaged prior to analysis.


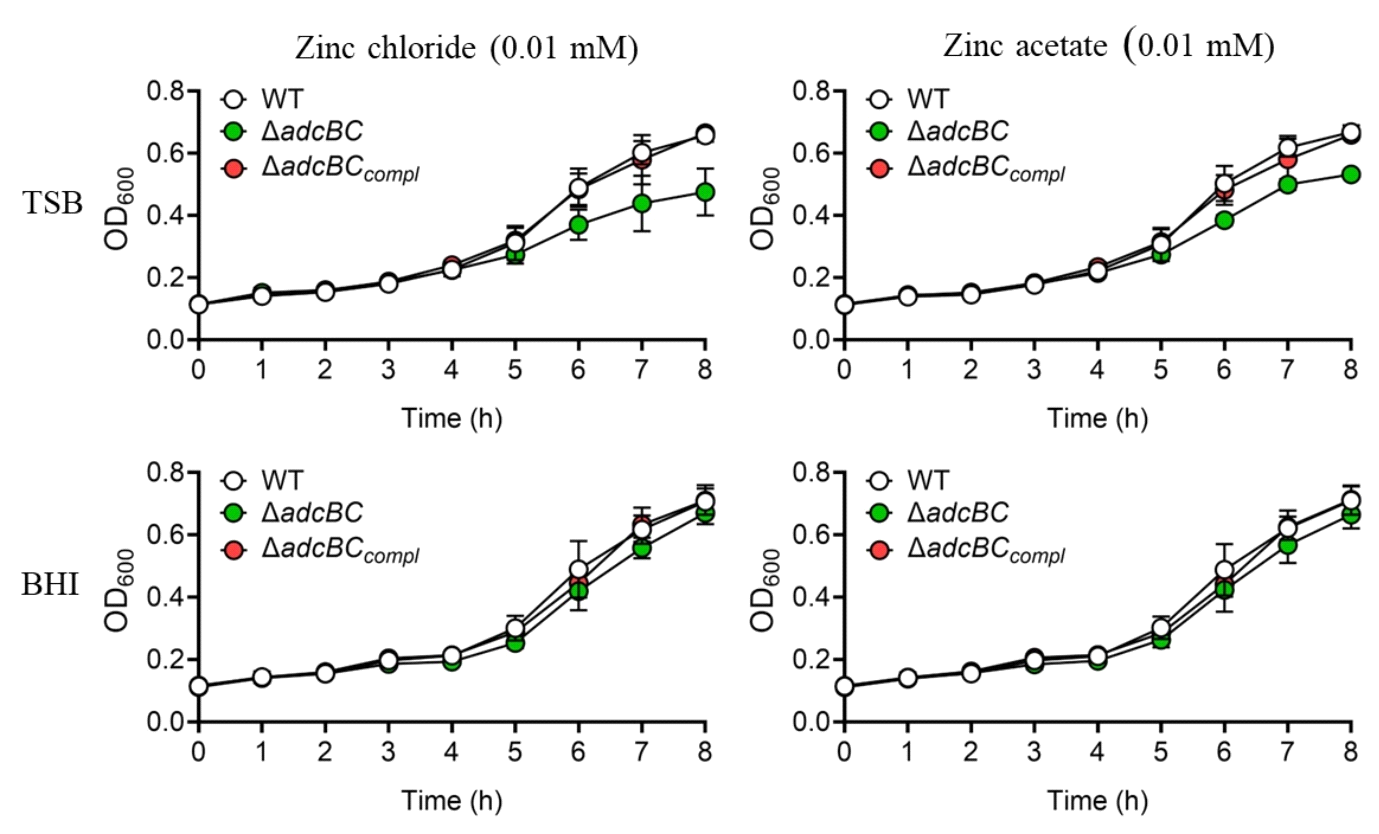


Fig. S2

**Effect of lactate on growth of *S. mutans* WT and Δ*adcBC* strains.** Growth curves of *S. mutans* UA159 wild-type (WT, left panel) and Δ*adcBC* mutant (right panel) cultured in BHI under untreated conditions or supplemented with sodium lactate (10 mM or 25 mM). Bacterial growth was monitored by measuring optical density at 600 nm (OD600) over 8 hours. Data represent mean ± SD from three independent biological replicates, each performed in technical duplicate.


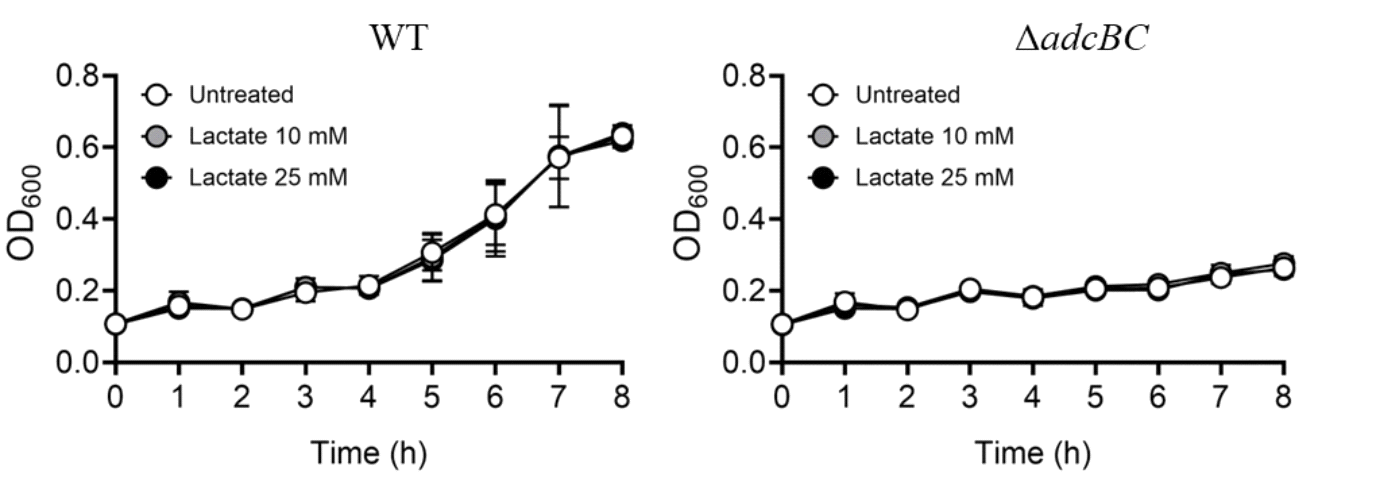

Supplement: Supplementary Material — Supplementary File.docx [file ZJOM_A_2691469_SM1553.docx]
